# A Novel Technique for a Successful Closed Reduction of a Bosworth Fracture-Dislocation of the Ankle

**DOI:** 10.7759/cureus.6632

**Published:** 2020-01-12

**Authors:** Juston Fan, Richard M Michelin, Ryne Jenkins, Minju Hwang, Michael French

**Affiliations:** 1 Orthopaedic Surgery, Riverside University Health System Medical Center, Moreno Valley, USA

**Keywords:** bosworth, ankle, fracture, closed reduction

## Abstract

The Bosworth fracture is defined as a bimalleolar fracture-dislocation of the ankle, with entrapment of the fibula behind the posterior tubercle of the distal tibia. In the current orthopedic literature, not only is this fracture pattern rare, but this type of fracture-dislocation has also been reported to be near impossible to close reduce, with the majority requiring early open reduction and internal fixation to prevent complications and poor clinical outcomes. Reported early complications include compartment syndrome and soft tissue complications from repeated closed reduction attempts. Complications associated with delayed operative intervention include post-traumatic adhesive capsulitis of the ankle and ankle stiffness. We present a case study of a 34-year-old male who sustained a Bosworth fracture-dislocation of the right ankle after a skateboarding accident. We describe a successful closed reduction performed in the emergency department, with a novel closed reduction technique. The patient tolerated the procedure well, with no complications. He was then scheduled for open reduction and internal fixation five days afterward, and upon post-operative follow-up, he recovered well with no complications. This technique focuses on reduction forces applied to the proximal fibular fragment, which is entrapped behind the posterolateral portion of the tibia. We believe that the key to successful reduction is applying an anterolateral/internal rotation force to this entrapped fragment. If successful, this fracture pattern may not require admission for compartment checks or early open reduction and internal fixation, thereby preventing complications and poor clinical outcomes. Our technique allows for a successful closed reduction of Bosworth fractures; however, further research exploring this reduction technique is warranted.

## Introduction

The Bosworth fracture is defined as a bimalleolar fracture-dislocation of the ankle, with entrapment of the fibula behind the posterior tubercle of the distal tibia, making closed reduction attempts largely unsuccessful [1]. Early open reduction with internal fixation is generally recommended to prevent serious complications. Bartonícek et al. described 108 cases of Bosworth fracture-dislocations in the literature, of which only 17 underwent successful closed reductions, whereas the remainder underwent early operative intervention. Of the 17 who underwent successful reductions, 5 were adults and 12 were pediatric patients [2]. The mechanism of injury is thought to be from an indirect force with axial loading on a supinated foot. The excessive amount of external rotation causes the talus to rotate 90 degrees from its anatomic position, and the increased force on the lateral collateral ligaments draws the fibula behind the posterior tibial tubercle. If there is sufficient force, the fibula breaks against the posterior tibial border and becomes entrapped [3]. On the anteroposterior (AP) radiograph, a Bosworth fracture will demonstrate the overlapping of the distal tibia and fibula, whereas the lateral view will demonstrate a posterior subluxation of the talus [2]. A pathognomonic term called the “axilla sign” in the AP mortise view will show the cortical density in the axilla (or shoulder) of the medial tibial plafond [4]. This is due to the internal rotation of the tibia, when the film alignment is based on a posteriorly displaced fibula relative to the tibia [2].

Early recognition of a Bosworth fracture-dislocation is crucial, as associated soft tissue damage and compartment syndrome have been reported. Delays in adequate treatment are linked with inferior clinical outcomes, with the majority requiring early open reduction and internal fixation [5-8]. We present a case report of a 34-year-old male who underwent a rare, successful closed reduction allowing for delayed open reduction with internal fixation without complication. We describe our closed reduction and surgical techniques with the support of radiographs.

## Case presentation

**Figure 1:**
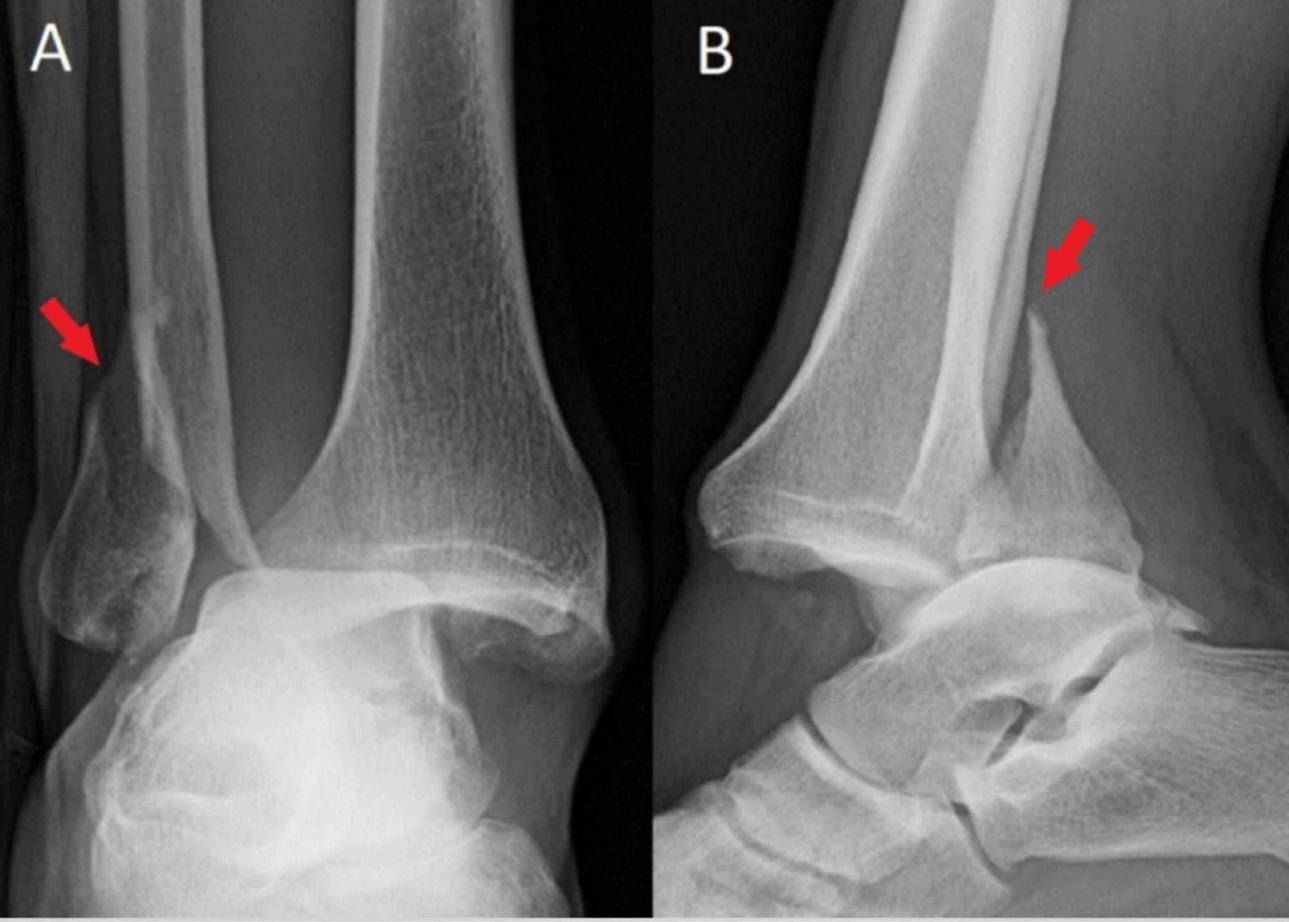
Initial injury radiographs Anteroposterior (A) and lateral (B) radiographs of the initial injury show tibiotalar dislocation and fracture of the lateral malleolus (red arrows). Initially it was difficult to assess the location of the proximal fragment of the fibula in relation to the posterior tibial tubercle due to the external rotation of the talus relative to the tibia.

A 34-year-old male presented to the emergency department following an external rotational injury of his right ankle after falling off his skateboard. He was unable to ambulate and had a notable deformity of the right ankle. AP, mortise, and lateral radiographs were obtained. Initial radiographs demonstrated a bimalleolar ankle fracture with tibiotalar dislocation (Figure 1).

Upon initial exam, there was tenderness to palpation about the ankle. Sensation to light touch was grossly intact in the deep and superficial peroneal, saphenous, sural, and medial and lateral plantar nerve distributions. Dorsalis pedis and posterior tibialis pulses were palpable and intact. There was an obvious deformity of the ankle, with the tibia prominently anterior to the talus. There were superficial anteromedial abrasions over the ankle but no other obvious open wounds or lacerations.

**Figure 2:**
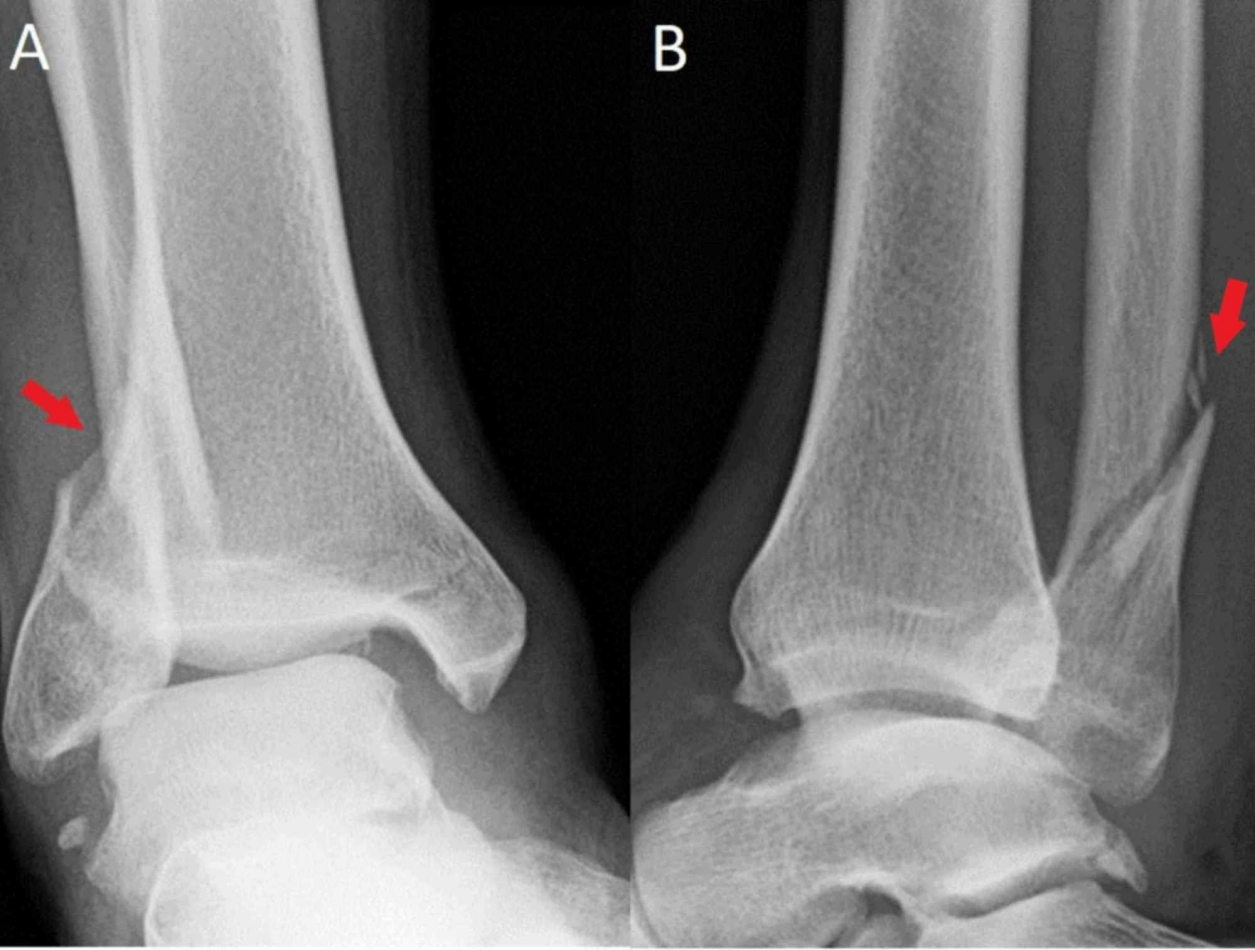
First attempted closed reduction Anteroposterior (A) and lateral (B) radiographs after first attempted closed reduction with notable unacceptable alignment and subluxation of the tibiotalar joint. These radiographs show clearly that the proximal fibular fragment is entrapped posterior to the tibia (red arrow).

**Figure 3:**
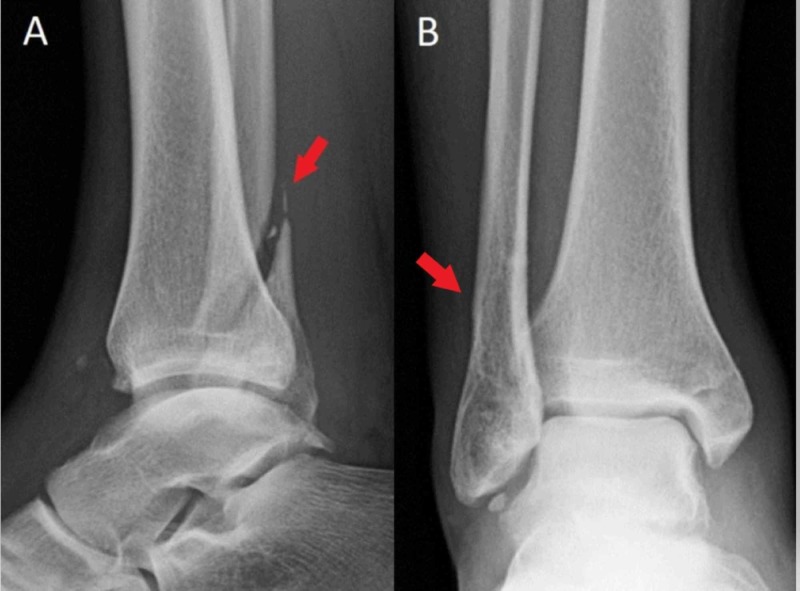
Successful closed reduction Lateral (A) and anteroposterior (B) radiographs demonstrate successful closed reduction after the described technique. Reduction of the tibiotalar joint with widening of the fibular fracture is shown (red arrows). Medial malleolar fracture remains minimally displaced. The patient was splinted after closed reduction.

The patient underwent conscious sedation with ketamine performed by the emergency department physicians. First attempted closed reduction involved standard bimalleolar ankle fracture-dislocation reduction techniques including accentuation of the injury and reduction of the tibiotalar joint with an anterior force applied to the foot with traction and dorsiflexion. After this failed attempt at a standard closed reduction technique, further assessment of radiographic imaging revealed posterior displacement of the proximal segment of the fibula behind the tibia, consistent with a Bosworth fracture-dislocation (Figure 2). At this point, a second closed reduction was attempted. The patient’s knee was flexed, and the ankle was put into dorsiflexion with traction. An anterior force was applied to the talus with supination and internal rotation of the foot. Another set of hands was required proximal to the fracture site of the fibula along the midshaft of the fibula. By palpating the fibular shaft proximally to distally, we were able to recognize the proximal fibular fragment displaced posteriorly behind the tibia. The key to our reduction technique involved placing our fingers along the posterior medial surface of the fibular shaft and applying a lateral and anterior force, while the other hand was placed along the tibial shaft to provide stabilization and a counter-force medially. A palpable reduction was achieved. Post-reduction films were obtained and showed adequate reduction (Figure 3), after which a three-sided plaster splint was applied. The patient was discharged home with non-weight-bearing status and scheduled for open reduction and internal fixation in five days from the date of injury.

**Figure 4:**
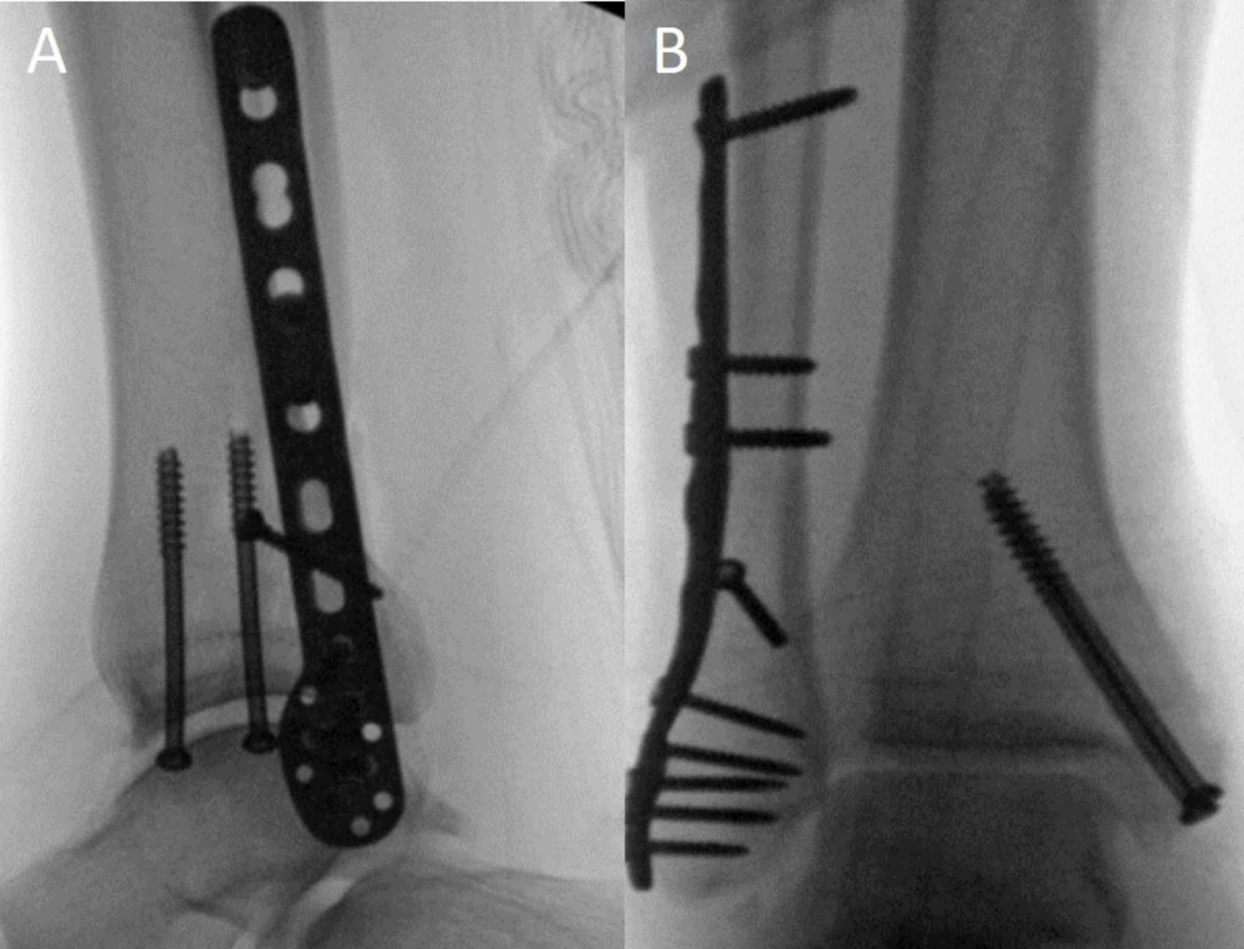
Post-operative fluoroscopic images Anteroposterior (A) and lateral (B) fluoroscopic images of open reduction and internal fixation five days after successful closed reduction and splinting. Two 4.0-mm partially-threaded cannulated lag screws were used for the medial malleolus, and a DePuy Synthes (Oberdorf, Switzerland) fibular locking plate with a 2.7-mm lag screw was used for the fibula fracture.

During the operative intervention, the patient was positioned and prepped in the supine position, with a bump under the ipsilateral hip. An incision was made over the lateral malleolus approximately 10 cm in length. The fracture was exposed and then anatomically reduced using serrated reduction forceps. A 2.7-mm screw was used to lag the fracture using standard AO technique. A one-third tubular neutralization plate was applied and fixated with a combination of 3.5-mm cortical and 2.7-mm locking screws. A 6-cm incision was made centered over the medial malleolus. Guidewires for cannulated screw fixation were placed in a diverging pattern. A cannulated drill was used to open the outer cortex of the medial malleolus, and 4.0-mm cannulated, partially threaded lag screws were placed across the fracture site. An external rotation stress test was performed, which demonstrated no loss in tibiofibular overlap and no medial clear space widening. Final fluoroscopic images were taken (Figure 4). Surgical incisions were closed and dressed. The patient was placed into a well-padded short leg splint with instructions to follow-up in the office two weeks post-operatively.

During the patient’s two-week follow-up, his incision sites were well healed and the sutures were removed at that time. He was then put in a short leg cast, with instructions to remain non-weight-bearing. The patient was again seen at six weeks post-operatively, in which his short leg cast was removed, and the patient was told to weight-bear as tolerated.

## Discussion

There have been only eight reported instances in literature of successful closed reduction of a Bosworth fracture in adults. This type of ankle fracture is challenging to reduce due to the posterolateral osseous portion of the tibia that prevents closed reduction of the fibula. In order to have successful outcomes, most cases require open reduction and internal fixation to achieve acceptable reduction and prevent associated complications such as compartment syndrome, skin complications, and an otherwise poor clinical outcome. The “axilla sign” is a radiographic marker that can help identify the Bosworth fracture-dislocation [4]. Bosworth’s original paper as well as subsequent literature articles cite the difficulty of this type of fracture pattern and the necessity for operative treatment [5]. To date, there has been no closed reduction technique described for this fracture pattern.

In the literature, Silverio et al. report a Bosworth fracture-dislocation, in which attempted closed reduction failed and the patient needed to be taken to the operating room for open reduction and internal fixation. During surgery, the distal fibula was approached using the standard lateral incision, where the fracture site was exposed and it was noted that the proximal aspect of the distal fibula was entrapped behind the posterolateral edge of the tibia. After successful open anatomic reduction, the fracture was stabilized with internal fixation, and the patient made a full recovery without complications after weight-bearing and return to normal activities at six weeks [9].

We present a novel technique for a successful closed reduction of a Bosworth fracture that focuses on reduction forces applied to the proximal fibular fragment that is entrapped behind the posterolateral portion of the tibia. Once enough force was applied to this piece with an anterolateral and internal rotation force, a successful closed reduction of this entrapment was achieved. Our case study reflects the success of our reduction technique in an individual with a body mass index of 23. Patients with a larger soft tissue envelope or heavy musculature surrounding the fracture may preclude the ability to palpate and manipulate the proximal fibular fragment, thereby preventing successful reduction. We recognize that our described technique may not be applicable to this subset of patients.

**Figure 5:**
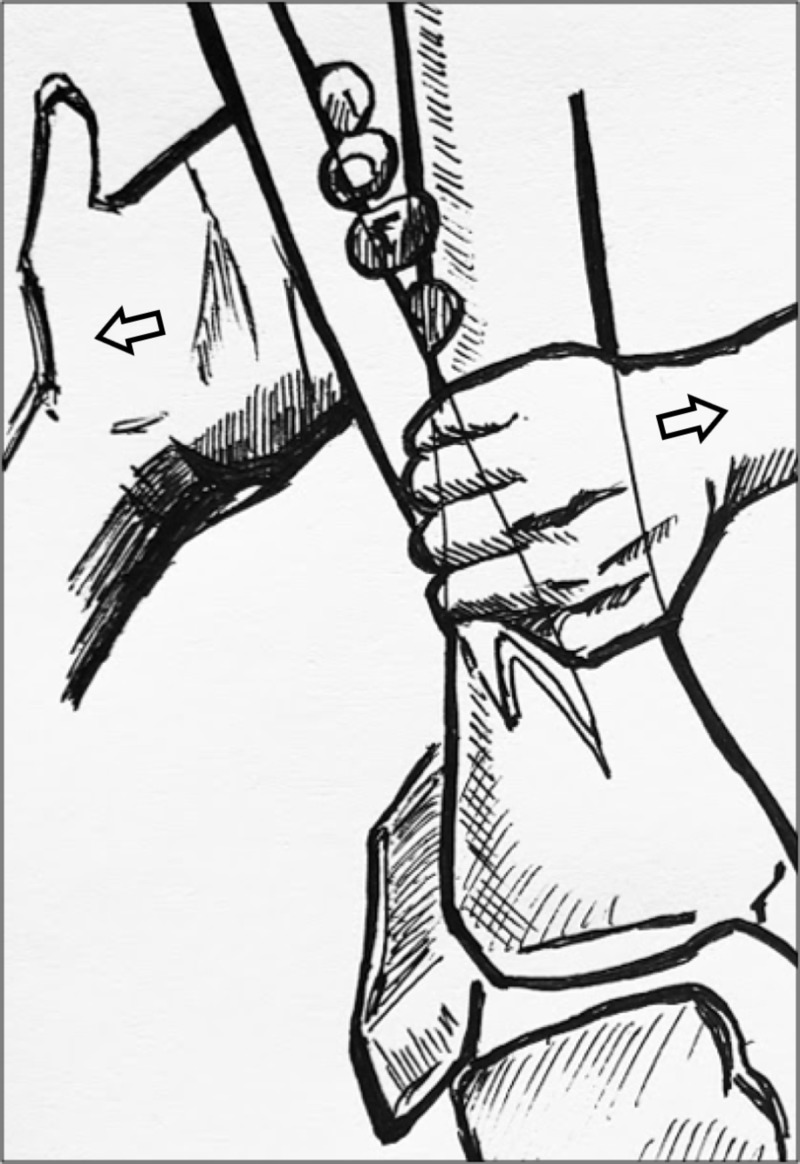
Visual representation of reduction technique A visual depiction of the reduction technique drawn by the authors. One hand is placed proximal to the fracture site, along the midshaft of the fibula, over the posterior medial surface of the fibular shaft, applying a lateral force with internal rotation. A second hand contacts the tibia directing a medial force. Arrows are in the direction of the force applied.

During our positioning of the patient during closed reduction, we kept the ankle in dorsiflexion, with attempts to stabilize the tibiotalar joint. We decided to attempt the reduction of the entrapped fibula while keeping the talus reduced. We recognize that this may have kept the soft tissues of the posterior compartment tightened, perhaps making reduction more difficult. By keeping the knee flexed, we believe we were able to balance the forces of the posterior compartment by decreasing the tension from the gastrocnemius musculature. This was well tolerated under standard conscious sedation in the emergency department. We believe there may be a possible merit to attempt plantar flexion of the ankle during reduction of the Bosworth fracture-dislocation, further decreasing the tension of the posterior musculature. Nonetheless, the primary focus of the reduction involves direct palpation and application of a lateral force to the proximal fibular fragment. We have drawn a visual depiction of this reduction technique (Figure 5). Alterations of our reduction technique, including changes in positioning of the ankle and foot, should be explored in the future as possible variations to our described technique.

The importance of recognizing this rare fracture-dislocation must be stressed, as compartment syndrome is a potential sequela of this condition [6]. Compartment syndrome may also occur from repeated failed reduction attempts, which may also cause iatrogenic soft tissue trauma [6]. Post-traumatic adhesive capsulitis has been a reported complication of failed closed reduction, with surgical intervention two to four days after the initial injury. Good outcomes were reported with operative fixation of the Bosworth fracture-dislocation within 24 hours of injury, with reduced ankle stiffness [7]. Additional imaging such as CT scan and three-dimensional CT reconstruction may allow for improved visualization and hastened diagnosis, as many cases remain undiagnosed as it is difficult to recognize this fracture pattern on radiographs. Early recognition and accuracy in diagnosis of this rare fracture pattern are crucial in maximizing satisfactory clinical outcomes and preventing associated complications. We present here a case report highlighting a novel closed reduction technique of a Bosworth fracture-dislocation allowing for delayed operative intervention on an outpatient basis without complications. Although there is a paucity of literature on this topic, we suggest that this fracture pattern may not require admission for compartment checks or prompt open reduction and internal fixation. Likely, this fracture pattern can be managed similarly to standard bimalleolar equivalent ankle fracture-dislocation if successful closed reduction is achieved. Future research comparing outcomes of successful closed reduction with delayed surgical intervention versus prompt open reduction with internal fixation is required.

## Conclusions

Our patient was discharged home from the emergency department after a successful closed reduction. However, there have been reports of compartment syndrome that bring up the concern for close compartment checks as an inpatient. Also, after a thorough literature review, we conclude that it may be prudent to urgently fix these Bosworth fracture-dislocations surgically within 24-48 hours to have better outcomes such as reduced risk of post-traumatic arthritis and reduced ankle stiffness. The Bosworth fracture-dislocation is described as nearly impossible to achieve close reduction and must be taken to the operating room to surgically correct this fracture. We propose a reasonable option to avoid urgent surgical intervention if successful closed reduction is achieved. Here we have described a novel technique for a successful closed reduction and delayed surgical intervention of a Bosworth fracture-dislocation that resulted in a satisfactory clinical outcome. We focus primarily on palpation and manipulation of the proximal fibular fragment away from the location of entrapment behind the posterior tibia. We also stress that early recognition of this rare fracture-dislocation pattern is essential in obtaining good outcomes and preventing soft tissue complications. Future studies are needed to examine the best methods of closed reduction and the outcomes of successful closed reduction versus early open reduction.

## References

[1] Bosworth DM (1947). Fracture-dislocation of the ankle with fixed displacement of the fibula behind the tibia. J Bone Joint Surg Am.

[2] Bartonícek J, Rammelt S, Kostlivýk K (2017). Bosworth fracture: a report of two atypical cases and literature review of 108 cases. Fuß & Sprunggelenk.

[3] Downey M, Motley T, Kosmopoulos V (2016). The Bosworth ankle fracture: a retrospective case series and literature review. EC Orthopaedics.

[4] Khan F, Borton D (2008). A constant radiological sign in Bosworth’s fracture: ‘‘the axilla sign’’. Foot Ankle Int.

[5] Hancock J (2015). Rare ankle fracture-pattern: Bosworth fracture can lead to post-traumatic arthritis if unrecognized early. MOJ Orthop Rheumatol.

[6] Beekman R, Watson JT (2003). Bosworth fracture-dislocation and resultant compartment syndrome: a case report. J Bone Joint Surg Am.

[7] Lui TH, Chan KB, Kong CC, Ngai WK (2008). Ankle stiffness after Bosworth fracture dislocation of the ankle. Arch Orthop Trauma Surg.

[8] Wright SE, Legg A, Davies MB (2012). A contemporary approach to the management of a Bosworth injury. Injury.

[9] Silverio A, Rebich E, Bass-Williams M, Vardiabasis N, Robinson M (2014). Bosworth ankle fracture-dislocation. The Orthopod.

